# Genetic associations between orexin genes and phenotypes related to behavioral regulation in humans, including substance use

**DOI:** 10.1038/s41380-025-02895-4

**Published:** 2025-01-29

**Authors:** Fazil Aliev, David De Sa Nogueira, Gary Aston-Jones, Danielle M. Dick

**Affiliations:** 1 Department of Psychiatry, Rutgers Robert Wood Johnson Medical School, Piscataway, NJ 08854 USA; 2 Rutgers Addiction Research Center, Brain Health Institute, Rutgers University and Rutgers Health, Piscataway, NJ 08854 USA

**Keywords:** Neuroscience, Genetics

## Abstract

The hypothalamic neuropeptide system of orexin (hypocretin) neurons provides projections throughout the neuraxis and has been linked to sleep regulation, feeding and motivation for salient rewards including drugs of abuse. However, relatively little has been done to examine genes associated with orexin signaling and specific behavioral phenotypes in humans. Here, we tested for association of twenty-seven genes involved in orexin signaling with behavioral phenotypes in humans. We tested the full gene set, functional subsets, and individual genes involved in orexin signaling. Our primary phenotype of interest was Externalizing, a composite factor comprised of behaviors and disorders associated with reward-seeking, motivation, and behavioral regulation. We also tested for association with additional phenotypes that have been related to orexin regulation in model organism studies, including alcohol consumption, problematic alcohol use, daytime sleepiness, insomnia, cigarettes per day, smoking initiation, and body mass index. The composite set of 27 genes corresponding to orexin function was highly associated with Externalizing, as well as with alcohol consumption, insomnia, cigarettes per day, smoking initiation and BMI. In addition, all gene subsets (except the *OXR2/HCRTR2* subset) were associated with Externalizing. BMI was significantly associated with all gene subsets. The “validated factors for *PPOX*/*HCRT*” and “*PPOX*/*HCRT* upregulation” gene subsets also were associated with alcohol consumption. Individually, 8 genes showed a strong association with Externalizing, 12 with BMI, 7 with smoking initiation, 3 with alcohol consumption, and 2 with problematic alcohol use, after correction for multiple testing. This study indicates that orexin genes are associated with multiple behaviors and disorders related to self-regulation in humans. This is consistent with prior work in animals that implicated orexin signaling in motivational activation induced by salient stimuli, and supports the hypothesis that orexin signaling is an important potential therapeutic target for numerous behavioral disorders.

## Introduction

Orexin neurons are located within the hypothalamus and produce two peptides, orexin-A and B (also known as hypocretin 1 and 2), both resulting from the cleavage of PPOX/HCRT (encoded by *PPOX*/*HCRT*) by a proprotein convertase subtilisin/kexin (*Pcsk*) [1, 2]. Orexin peptides signal through two G-protein coupled receptors, orexin receptor 1 and 2 (OXR1/2; also known as hypocretin receptor 1 and 2) which are expressed abundantly throughout the central and peripheral nervous systems [3] (for review see Gotter et al. [4]).

The brain orexin system plays a central role in motivation, impulsive action and behavioral disinhibition [5–7]. Initital evidence in the orexin field revealed a major role for orexin in feeding. Orexin peptides increased upon fasting, and a selective OXR1 antagonist reduced food intake in rats [1, 8, 9]. There is also extensive literature revealing a role for the orexin sytem in behavioral dishinibition associated with high motivation for salient rewards [10] and impulsive actions [11] as associated with substance use. OXR1 blockade attenuated cocaine-seeking and demand [6, 12, 13], motivation for alcohol as well as relapse to alcohol seeking [7, 14–17], and motivation for nicotine [18], as well as for opioids [19–21]. Moreover, selective OXR1 or dual OXR1/2 antagonists attenuated cocaine-associated impulsivity [11, 22].

Orexin signaling has also been strongly implicated in sleep-wake regulation. Ablation of orexin neurons in mice led to a narcolepsy-like phenotype as in humans [23, 24], and hypophagia [25, 26]. Loss of the OXR2 in dogs produced a strong narcolepsy-like phenotype [27], and antagonism of OXR1/2 promoted sleep in humans [28, 29]. Also, a missense variant in the cleavage site of *PPOX* has been associated with idiopathic hypersomnia in humans [30].

PPOX expression is under the influence of several transcription factors. *Foxa2, Nr6a1, Ebf2, Plagl1, Lhx9, Nptx2b* have all been described previously as enhancers of PPOX expression [31–36], and *Igfbp3*, *Bhle41* and *Tnf* dampen PPOX expression [37–39]. Several of these genes have been associated with sleep or feeding disturbances. In humans, variants within *BHLE41* induced a short sleep phenotype and resistance to sleep deprivation [40, 41]. Overexpression of *Nptx2b*, upregulator, promoted restistance to sleep in zebrafish [31] whereas overexpression of *Igfbp3*, downregulator, increased sleep in mice [37]. Likewise, ablation of either *Lxh9* or *Ebf2*, both upregulators, promoted a narcoleptic phenotype [32, 33]. Overexpression of *Foxa2* leads to hyperphagia in mice [34]. Other exploratory studies revealed several identity marker genes for orexin neurons such as *Scg2, Cbln1, Slc2a13, Peg3, Ldb2, Six6, Nr2f2, Prrx1, Nkx6-2, Ahr*, and *Vgf* [42, 43]. Some of these genes might act as transcription factors but such a role has not been confirmed.

In the present study, we examined associations of human genes involved in the regulation, cleavage or transport of orexin with a number of phenotypes related to self-regulation and sleep impairment, common features of individuals suffering from substance use disorder [44–46].

## Materials and methods

We analyzed genotypic data from genome-wide association study (GWAS) data available on eight phenotypes with well-powered human GWAS data available, enabling us to test for associations between orexin genes and human self-regulatory outcomes. Our primary outcome of interest was *Externalizing*, a term used in clinical psychology to refer to a constellation of disorders and behaviors that share an element of behavioral dysregulation. Externalizing outcomes are highly comorbid and twin-family data have robustly demonstrated that there is a shared set of underlying genetic risk factors that broadly impact outcomes related to behavioral undercontrol [47–49]. Given the role of orexin signaling in reward seeking, motivation and impusivity (described above), we believe that the orexin gene family is a strong candidate for influencing the externalizing phenotype. We also tested several additional phenotypes for which well-powered GWAS data were available to conduct gene set analyses for associations with behavioral phenotypes that have been reported for orexin in model organisms, including alcohol consumption, problematic alcohol use, daytime sleepiness, insomnia, cigarettes per day, smoking initiation and body mass index (BMI). Details of discovery GWAS including samples used in meta-analyses are presented in Table 1 and described further below:

**Table 1:** Summary of GWAS summary statistics.

*Phenotype*	*Cohort: Original Phenotypes*	*Max N*	*h*^2^ (*SE*)	Mean χ^2^	LDSC Intercept	Reference
Externalizing	UKB + PGC: problem alcohol use	1,492,085	0.061 (*0.002*)	3.114	1.115	Karlsson Linnér et al., [56]
	PGC: ADHD					
	ICC: lifetime cannabis use					
	GSCAN: lifetime smoking initiation					
	SSGAC: number of sexual partners					
	SSGAC: age of first sexual intercourse					
	SSGAC: general risk tolerance					
Alcohol consumption	GSCAN: drinks per week	738,029	0.048 (*0.002*)	1.56	0.941	Kranzler et al., [56]; Liu et al., [57]
	MVP: AUDIT-C					
Problematic Alcohol Use	PGC: alcohol dependence	433,000	0.051 (*0.003*)	1.396	1.021	(Kranzler et al., [56]; Sanchez-Roige et al., [53]; Walters et al., [54])
	MVP: AUD					
	UKB: AUDIT-P					
Daytime Sleepeness	UKB EUR: Excessive Daytime Sleepeness	452,071	0.069 (*0.01*)	1.14	1.02	Wang et al., [61]
Insomnia	UKB EUR: Insomnia	453,379	0.066 (*0.029*)	1.19	1.00	Lane et al., [62]
Cigarettes per Day	Meta analysis of 60 cohorts; CPD	327,000	0.08 (*0.005*)	2.078	1.097	Saunders et al., [63]
Smoking Initiation	Meta analysis of 60 cohorts; Smoking Initiation	805,000	0.08 (*0.002*)	6.054	1.44	Saunders et al., [63]
BMI	Locke et al. meta analysis: BMI	700,000	0.22 (*0.03*)	3.9	1.03	Yengo et al., [65]; Locke et al., [64]
	UKB: BMI					

The statistics reported in this table were all estimated with LD Score regression (LDSC) with heritability (*h*^2^) is on the observed scale. LDSC Intercept is the estimated LD Score regression intercept, Mean χ^2^- mean chi-square value of the GWAS SNPs left for LDSC regression after QC filtering. GSCAN = GWAS and Sequencing Consortium of Alcohol and Nicotine Use; *MVP* million veterans program, *PGC* psychiatric genomics consortium, *UKB* UK biobank, *ICC* international cannabis consortium, *SSGAC* social science genetic association consortium, *AUDIT-C* alcohol use disorder identification test consumption scale, *AUDIT-P* alcohol use disorder identification test problem scale, *PAU* problematic alcohol use, *ADHD* attention-deficit hyperactivity disorder, *CPD* cigarettes per day, *BMI* body mass index.

### Externalizing

Externalizing is an umbrella term used to describe a variety of behaviors/problems related to behavioral disinhibition that are correlated at the phenotypic and genetic levels [50]. We derived our genetic data for externalizing from a recent multivariate, common factor GWAS [51]. The latent externalizing factor included seven GWAS related to behavioral undercontrol as inputs: ADHD (N = 53,293) [52], problematic alcohol use (N = 164,121) [53, 54], lifetime cannabis use (N = 186,875) [55], age at first sexual intercourse (N = 357,187) [56], number of sexual partners (N = 336,379) [56], general risk tolerance (426,379) [56], and lifetime smoking initiation (N = 1,251,809) [57]. The estimated sample size for the externalizing factor was approximately 1.5 million individuals.

### Alcohol consumption

We meta analyzed two publicly available large scale GWAS of alcohol consumption. The first dataset comes from the GWAS & Sequencing Consortium of Alcohol and Nicotine use (GSCAN) analysis of drinks per week (without the 23&me sub-sample) in approximately 550 K individuals of European ancestries [57]. The second GWAS comes from the Million Veterans Program (MVP) GWAS of the first three items related to alcohol consumption in of the Alcohol Use Disorder Identification Test (AUDIT), referred to as AUDIT-C, in approximately 200 K individuals of European ancestries [58]. These sets of summary statistics were highly correlated (*r*_*g*_ = 0.70), and we used a sample-size weighted meta-analysis in METAL [59], resulting in a total sample size of ~750 K individuals.

### Problematic alcohol use (PAU)

We created GWAS data for PAU by meta-analyzing three sets of summary statistics: PAU as measured in the Million Veterans Program (phase 1: N~202 K, phase 2: 65.4 K) [58, 60], Alcohol Dependence as analyzed by the Psychiatric Genomics Consortium (N~46.5 K) [54], and the problem subscale of the AUDIT analyzed in the UK Biobank (AUDIT-P, N~121.5 K) [53], again using a sample-size weighted meta-analysis because of the relatively strong genetic overlap between each (*r*_*g*_ = 0.60–1.00). Our final sample size was N~435 K individuals of European ancestries.

### Daytime sleepiness

Excessive daytime sleepiness is a chief symptom of chronic insufficient sleep as well as of several primary sleep disorders, such as sleep apnea, narcolepsy, and circadian rhythm disorders. We used a GWAS of 452,071 individuals from the UK Biobank. In the UK Biobank participants of European genetic ancestry self-reported the frequency of daytime sleepiness using the question: “How likely are you to doze off or fall asleep during the daytime when you don’t mean to? (e.g.: when working, reading or driving)”, with the answer categories “never” (N = 347,285), “sometimes” (N = 92,794), “often” (N = 11,963), or “all of the time” (N = 29) [61].

### Insomnia

UK Biobank participants of European ancestry (n = 453,379) self-reported insomnia symptoms to the question “do you have trouble falling asleep at night or do you wake up in the middle of the night?”. In this sample, 29% of individuals self-reported frequent insomnia symptoms (“usually”), with a higher prevalence in women (32 vs. 24%) and in older participants, shift workers, and those with shorter self-reported sleep duration. GWAS of any insomnia symptoms (“never/rarely” vs. “sometimes”/”usually” insomnia symptoms, n = 345,022 cases and 108,357 controls), adjusting for age and sex was used in our analyses [62].

### Cigarettes per day and smoking initiation

We used summary statistics from a meta-analysis of GWAS data from 60 European ancestry cohorts from the GSCAN II study [63]. Amount smoked among current and former regular smokers was measured as cigarettes smoked per day (CigDay; n = 326,497). Smoking initiation was measured by asking whether an individual ever smoked regularly (smoking initiation (SmkInit); n = 805,431 (393,707 cases)).

### Body mass index (BMI)

The BMI meta-analysis combined results from meta-analyses of 250,000 individuals [64] and 450,000 UKB individuals of EUR ancestry totaling n = 700,000 [65]. BMI measured or self-reported weight in kg per height in meters squared was adjusted for age, age squared, and any necessary study-specific covariates (for example, genotype-derived principal components) in a linear regression model.

### Gene-set analyses

Our primary analyses were gene-set analyses that tested for overall association with the set of 27 orexin genes (*FOXA2*, *NR6A1*, *EBF2*, *PLAGL1*, *IGFBP3*, *BHLHE41*, *LHX9*, *SCG2*, *CBLN1*, *SLC2A13*, *PEG3*, *LDB2*, *SIX6*, *NR2F2*, *PRRX1*, *PCSK1*, *NPTX2*, *TNF*, *NKX6-2*, *NKX2*-1, *AHR*, *HCRTR1*, *HCRTR2*, *SIRT1*, *ARNTL*, *VGF*, *HCRT*) and the 8 outcomes. We created a gene-set containing all 27 genes, and 6 functional gene subsets: Subset “validated factors for *PPOX*/*HCRT*” represents transcription factors reported as functional regulators for *PPOX*/*HCRT* expression [31–39]; subset “predicted factors for *PPOX*/*HCRT*” represents transcription factors reported as putative regulators from single-cell data [42, 43]; subset “*PPOX*/*HCRT* upregulation” includes genes reported as *PPOX*/*HCRT* upregulators [31–36]; subset “*PPOX*/*HCRT* downregulation” includes genes reported as *PPOX*/*HCRT* downregulators [37–39]; subset “*OXR2*/*HCRT2*” includes genes described as *Oxr2* transcription factors [66–68]; subset “orexin transport” includes genes involved in transport and cleaving of *PPOX*/*HCRT* [30, 69].

We conducted gene-set analyses using the *R* COMBined Association Test (COMBAT) package [70]. COMBAT uses the SNP level p-values from GWAS data and correlations between SNPs from ancestry-matched samples and performs an extended Simes procedure to combine multiple parallel association test results performed by using Gates, Vegas (five Vegas tests and combined test) and simpleM methods and creates an overall association p-value. Compared to individual tests, COMBAT shows higher overall performance and robustness across a wide range of genetic models. Genetic results for the eight phenotypes were tested with all seven orexin gene sets including the full set. We corrected COMBAT p-values for multiple testing using Bonferroni correction by multiplying p-values by the total number of analyses n = 56 (eight phenotypes and seven orexin gene sets, including the full set). This represents a conservative correction because several of the phenotypes and gene sets were correlated.

### Individual gene analyses

We also performed individual gene-based tests with each of the 27 genes separately. We first performed MAGMA gene-based analysis on the GWAS input data through FUMA [71] using the externalizing GWAS results, followed by the secondary outcomes of interest. FUMA uses input GWAS summary statistics to compute gene-based *P*-values (gene analysis). The gene-based *P* value was computed for protein-coding genes by mapping SNPs to genes if SNPs are located within the genes. GWAS results from each of the samples were loaded to FUMA SNP2GENE software for gene-based analyses. In SNP2GENE SNPs are annotated with their biological functionality and mapped to genes based on positional, eQTL and chromatin interaction information. FUMA first defines independent significant SNPs depending on LD structure and defines genomic risk loci and then annotates for functional consequences on gene functions. As FUMA corrects for the number of genes used in analyses we corrected p-values for multiple testing using Bonferroni correction by multiplying by eight, which is the number of GWAS phenotypes used in analyses.

## Results

For each of the gene sets and each of the phenotypes we created a subset of SNPs located in all genes of the set from the GWAS results and provided that input to COMBAT. Table 2 shows the results for all COMBAT analyses. P values created by COMBAT for gene sets are corrected for multiple testing and significant P-values are bolded in the table. Externalizing was highly associated with the full orexin gene set and all functional subsets, except the *OXR2x2*/*HCRTR2* set. Additionally, the full orexin gene set was highly associated with alcohol consumption as well as with insomnia, cigarettes per day, smoking initiation and BMI, after correcting for multiple tests. “Validated Factors for *PPOX*/*HCRT*” and “*PPOX* / *HCRT* upregulation” subsets were associated with alcohol consumption and BMI. BMI was significantly associated with all gene sets. The “predicted factors for *PPOX* / *HCRT*” gene set showed significant association with cigarettes per day, smoking initiation and BMI along with Externalizing. Problematic alcohol use was only associated with the “*PPOX* / *HCRT* downregulation” gene set and daytime sleepiness was only associated with the “*OXR2*/*HCRTR2*” subset. We also observed an association between the “orexin transport” gene set with smoking initiation along with Externalizing and BMI.

**Table 2:** P values from gene set analyses.

	All genes combined	Validated factors for PPOX/HCRT	Ppox/Hcrt upregulation	Ppox/Hcrt downregulation	Predicted factors for PPOX/HCRT	Orexin transport	OxR2/Hcrtr2
Externalizing	**0.00012**	**0.00020**	**0.00018**	**0.00053**	**0.04250**	**0.00024**	0.73760
Alcohol Consumption	**0.00012**	**0.00052**	**0.00012**	1	0.21800	1	1
Problematic Alcohol Use	1	1	1	**0.02480**	1	1	1
Daytime Sleepiness	1	1	1	1	1	1	**<0.00001**
Insomnia	**0.00012**	1	1	1	1	1	0.232
Cigarettes per Day	**0.01160**	1	1	1	**0.00906**	1	1
Smoking Initiation	**0.00043**	0.63740	0.70430	0.25930	**0.00035**	**0.02310**	0.08930
Body Mass Index	**<0.00001**	**0.00011**	**0.00011**	**0.00012**	**0.00011**	**<0.00001**	**<0.00001**

P values from COMBAT gene set analyses.

**Gene Set Contents: All genes combined** (FOXA2, NR6A1, EBF2, PLAGL1, LHX9, NPTX2, IGFBP3, BHLHE41, TNF, CBLN1, SLC2A13, PEG3, LDB2, SIX6, NR2F2, PRRX1, NKX6-2, AHR, VGF, SCG2, PCSK1, NKX2-1, HCRTR2, SIRT1, ARNTL, HCRTR1, HCRT); **Validated Factors for PPOX/HCRT** (FOXA2, NR6A1, EBF2, PLAGL1, LHX9, NPTX2, IGFBP3, BHLHE41, TNF); **Ppox/Hcrt upregulation** (FOXA2, NR6A1, EBF2, PLAGL1, LHX9, NPTX2); **Ppox/Hcrt downregulation** (IGFBP3, BHLHE41, TNF); **Predicted factors for PPOX/HCRT** (CBLN1, SLC2A13, PEG3, LDB2, SIX6, NR2F2, PRRX1, NKX6-2, AHR, VGF, SCG2); **Orexin Transport** (SGC2, PCSK1); **OxR2/Hcrtr2** (NKX2-1, HCRTR2, SIRT1, ARNTL).

The bold values indicate statistically significant findings as defined by p < .05.

Supplementary Table 3 presents the results from gene-based FUMA analyses. The *BHLHE41*, LHX9, *LDB2*, *NR2F2*, *PCSK1*, *TNF*, *HCRTR1* and *ARNTL* genes showed significant association signals with Externalizing after correcting for multiple tests. *NR6A1*, *LDB2* and *TNF* genes showed significant association signals with alcohol consumption. *BHLHE41* and *TNF* were associated with problematic alcohol use. *TNF* was significantly associated with Externalizing, alcohol consumption, problematic alcohol use and BMI. Twelve genes were associated with BMI. We found seven genes associated with smoking initiation. Only *LDB2* was associated with cigarettes per day. *NR2F2* was associated with both daytime sleepiness and insomnia. None of genes associated with smoking initiation was significantly associated with cigarettes per day.

## Discussion

Multiple lines of evidence implicate orexin signaling in behaviors related to dysregulation, including alcohol-related phenotypes, sleep disturbances, nicotine use and BMI. In this paper, we expand on this literature by demonstrating for the first time that common variants in genes related to the orexin system are involved in human phenotypes related to behavioral regulation, paralleling the support for involvement of the orexin system in the animal literature.

In model organism studies, orexin expression as well as orexin neuronal activation are increased following ethanol [16, 72, 73] or nicotine intake [74–77], whereas orexin antagonists attenuate consumption [15, 16, 18, 78, 79] as well as seeking of both alcohol and nicotine [16, 77, 80–82].

As noted above, orexin neurons regulate not only food intake and energy expenditure but also states of sleep and wakefulness resulting in a complex role in body weight. For example, loss of orexin function promotes narcolepsy-cataplexy as well as increased body weight in narcoleptic patients [83]. Conversely, orexin release maintains wakefulness and increased orexin levels correlate with weigh reduction in adolescents [84], indicating that increased orexin signaling promotes obesity resistance (for review on the relationships between orexin and body weight, see Sakurai [85], Perez-Leighton et al. [86], Mahoney et al. [87]).

Here we tested for associations between genes involved in the expression, cleavage or transport of PPOX or its receptors with the array of phenotypes examined. When tested as a set, we found that the 27 genes were significantly associated with Externalizing, alcohol consumption, cigarettes per day, smoking initiation, insomnia, and BMI, essentially mirroring the diversity of phenotype with which orexin is associated in model organism studies. We also found that all subsets of genes, differentiated by function, were significantly associated with Externalizing (except the *OXR2/HXRTR2* subset) and BMI. Gene variance, DNA methylation (DNAm) changes or decreased expression of *PCSK1*, *AHR*, *ARNTL*, *PEG3*, *PLAGL1*, and *TNFα* were previously associated with body weight and food intake in humans [88–95]. The association of these genes with BMI and food intake indicates that other genes from our study associated with BMI and food intake may also be of interest.

Individual genes that are involved in orexin regulation were also significantly associated with several of the component phenotypes investigated here. Regarding alcohol-related phenotypes, we noticed a striking association between *TNFα* variation, a *PPOX* downregulator, with Externalizing, alcohol consumption and problematic alcohol use. Interestingly, TNFα serum levels have been previously described as elevated in patients with alcohol use disorder [96–100] with higher concentrations in patients with liver disease. Moreover, the *TNF* gene polymorphism is associated with liver disease [101, 102], and two meta-analyses indicate the *TNF*-238 polymorphism is significantly associated with alcoholic liver disease [103] and alcohol dependence/abuse [104]. Other notable associations include *BHLHE41*, an orexin downregulator, with Externalizing and problematic alcohol use as well as *LDB2*, a predicted factor, with Externalizing and alcohol consumption. A previous study indicated an association between *BHLHE41* variants and PAU [105], consistent with comorbidity between PAU and circadian clock disturbances in humans [106]. However, there are no reports of alcohol-induced changes in *BHLHE41* gene expression. Alcohol consumption in humans is also linked to *LDB2* DNAm in blood cells [107]. DNAm levels might also be involved in *Ldb2* gene expression in PFC of mice following chronic ethanol exposure [108]. Altogether, our findings are consistent with previous reports of gene-phenotype associations, indicating that other individual associations may need further investigation.

One consideration of our study is that the orexin-related genes we examined are also involved with regulation of additional genes or neuropeptides. For example, *Foxa2*, *Plagl1* and *Scg2*, have been shown to regulate melanin-concentrating hormone (MCH) expression or transport, while *Peg3* and *Ldb2* are predicted transcritption factors for MCH. However, the majority of genes included in our analysis are either involved in peripheral processes (hepatogenesis, development) or with other types of neurons (dopamine, neuropeptide Y, pro-opio-melanocortin). Notably, our examination of the literature revealed that orexin is the only common target of the group of 27 genes, or of any of the subsets of genes, examined here.

We note that we were unable to test for any differential effects as a function of sex or age. This is a limitation of most human genetic association studies, which necessitate large sample sizes to have sufficient power to detect effects, and accordingly, combine all available data across multiple cohorts. The majority of the outcomes studied here were based on results from meta-analyses that corrected for age and sex, and did not report demographics for the samples or differentiated results, precluding our ability to test for moderating effects of sex or age. This will be an important future direction for human association studies.

### “Validated factors” subset

The “Validated factors” subset (*FOXA2*, *NR6A1*, *EBF2*, *PLAGL1*, *LHX9*, *NPTX2B*, *IGFBP3*, *BHLHE41*, *TNF*) is significantly associated with Externalizing and alcohol consumption, a result we expected given the involvement of the orexin system in behavioral dishinbition and motivation towards alcohol [7, 10, 109]. As this subset include factors that both up- or downregulate *PPOX* gene expression, we discuss these genes separately in the “*PPOX/HCRT upregulation” and “PPOX/HCRT downregulation” subsets* below.

### “Predicted factors” subset

The “predicted factors” subset (*SCG2, CBLN1, SLC2A13, PEG3, LDB2, SIX6, NR2F2, PRRX1, NKX6-2, AHR, VGF*) is significantly associated with Externalizing, cigarettes per day and smoking initiation. Previous reports indicated that *SLC2A13*, *BHLHE41*, *VGF* and *AHR* variants are associated with heavy and problem drinking in patients [105, 110] as well as with nicotine dependence in tobacco users [111]. DNAm levels of *NR2F2*, *PRRX1* and *NKX6-2* are increased in smokers [112, 113] rodents chronically exposed to nicotine [114]. Alcohol consumption is also linked to DNAm alterations in *LDB2*, *PEG3* and *NKX6-2* in human blood cells [107, 115, 116] or PFC in rodents [108]. These DNAm levels changes might be due to prenatal exposure to alcohol [117, 118]. Indeed, several reports indicated that paternal exposure to either alcohol [119–121] or nicotine [122, 123] impacted DNAm or mRNA levels of *Peg3*, Nrf2 and Nkx6-2 in offspring. Thus, several of the genes belonging to the “predicted factors” subset are modulated on the DNAm level, indicating that more epigenetic research on these genes is needed.

### “PPOX/HCRT upregulation” subset

The “PPOX/HCRT upregulation” subset (*FOXA2, NR6A1, EBF2, PLAGL1, LHX9, NPTX2B*) was significantly associated with alcohol consumption and Externalizing. To our knowledge, there are no studies investigating the impact of this gene subset on behavioral response to alcohol. However, several lines of evidence indicate that alcohol exposure can impact expression of these genes. An acute exposure to ethanol in rats increased mRNA levels of *Nptx2b* [124] whereas chronic alcohol increased expression of the *Ebf2* transcript in the dorsal hippocampus in mice [108]. *Nr6a1* is the target of several miRNAs in mice sperm following chronic ethanol inhalation [125] and Nr6a1 mRNA levels are regulated in the mPFC following alcohol abstinence in rats [126]. Altogether, these findings indicate that alcohol intake increases TF expression which may upregulate ppOx gene expression. These processes might underlie the orexin cell number increase in rats [72, 127] or humans [73] following alcohol exposure.

Several studies have found epigenetic alterations such as histone modifications or DNAm level changes in these genes, indicating that the epigenome also may be involved in these gene-behavior relationships. For example, ethanol exposure increased enrichment of the H3K27me3 histone mark (gene expression repressor) associated with a reduction of Plagl1 gene expression [128]. In addition, prenatal ethanol exposure impacted both expression and DNAm levels of *Plagl1* in mice [117], and maternal alcohol intake correlated with *PLAGL1* DNAm levels in newborns [129]. In most cases, a highly methylated promoter region is associated with a lower gene expression [130]. Therefore, these epigenetic regulations indicate a possible decrease of *PLAGL1* gene expression following alcohol exposure, and thus a decrease of PPOX expression, in contrast to reports above.

### “PPOX/HCRT downregulation” subset

The gene subset “*PPOX*/*HCRT* downregulation” (*IGFBP3, BHLHE41, TNF*) is significantly associated with problematic alcohol use and Externalizing. We discussed above the relationship between *BHLE41* and *TNFα* with PAU and Externalizing. Briefly, TNFα levels are higher in alcoholic patients [96–100]. Other studies reveal increased IGFBP3 concentrations in postmenopausal women following daily alcohol consumption [131] as well as increased IGFBP3 levels in both serum and liver tissue from patients with alcoholic liver disease [132]. These findings may indicate that TNFα and IGFBP3 levels increase in alcoholic patients and therefore decrease orexin expression. However, these elevated levels might be related to alcohol-induced steatohepatitis given the other roles of TNFα [133] and IGFBP3 [132] in the liver.

### “OXR2/HCRTR2” subset

As ORX2 is linked to the control of the sleep/wake cycle (for review, see Sun et al. [134]), we expected an association between this subset and sleep phenotypes. We observed a significant association between the ORX2 subset “*OXR2*/*HCRTR2*” (*NKX2-1, HCRTR2, SIRT1, ARNTL*) and daytime sleepiness. *Sirt1* expression is considerably decreased in sleep deprived rats [135]. Similarly, *Sirt1* expression deficiency impairs wakefulness [136]. In contrast, *Sirt1* overexpression improves sleep quality, an effect mediated through the regulation of the *Sirt1*/*Nkx2-1*/*Oxr2* pathway [67]. We did not find an association between the “*OXR2*/*HCRTR2*” subset and Insomnia. These findings indicate that SNPs impacting *OXR2* and its regulating genes may be more involved in the ability to sleep rather than simply in maintaining wakefulness.

### Orexin transport

The “Orexin transport” subset (*SGC2, PCSK1*) is significantly associated with problematic alcohol use and smoking initiation. Chronic nicotine exposure differentially regulates *Scg2* gene expression in animal models [137, 138] and *SCG2* gene expression increases in patients with a history of tobacco use [103]. Altogether, orexin transport activity and its aggregation in secretary granules [69] might be increased in patients with alcohol and tobacco use resulting in increased orexin release.

### Conclusions

Our data indicate that the genetic regulation of orexin signaling in humans is involved in several behavioral outcomes that have been associated with orexin function in animal studies, particularly in behaviors associated with an Externalizing phenotype that is characterized by elevated impulsivity and motivational activation [10]. These results support our view that interventions to modulate orexin signaling have great promise in treating several neuropsychiatric conditions, including those characterized by impulsivity and heightened motivation such as substance use disorders.

## Supplementary information

Supplementary table

## Data Availability

The data that support the findings of this study were publicly available genome-wide association study summary statistics, which are available from the authors or online repositories of the respective publications.
